# 4D‐CT deformable image registration using unsupervised recursive cascaded full‐resolution residual networks

**DOI:** 10.1002/btm2.10587

**Published:** 2023-08-22

**Authors:** Lei Xu, Ping Jiang, Tiffany Tsui, Junyan Liu, Xiping Zhang, Lequan Yu, Tianye Niu

**Affiliations:** ^1^ Department of Radiation Oncology the First Affiliated Hospital of Xi'an Jiaotong University Xi'an Shaanxi China; ^2^ Institute of Biomedical Engineering Shenzhen Bay Laboratory Shenzhen Guangdong China; ^3^ Department of Radiation Oncology Peking University 3rd Hospital Beijing China; ^4^ Loyola University Medical Center Maywood Illinois USA; ^5^ Department of Radiation Oncology Stanford University School of Medicine Stanford California USA; ^6^ Department of Radiation Oncology Ozarks Healthcare West Plains Missouri USA; ^7^ Department of Statistics and Actuarial Science The University of Hong Kong, HKSAR Hong Kong China; ^8^ Peking University Aerospace School of Clinical Medicine, Aerospace Center Hospital Beijing China

**Keywords:** 4D‐CT, deformable image registration, full‐resolution residual networks, unsupervised learning

## Abstract

A novel recursive cascaded full‐resolution residual network (RCFRR‐Net) for abdominal four‐dimensional computed tomography (4D‐CT) image registration was proposed. The entire network was end‐to‐end and trained in the unsupervised approach, which meant that the deformation vector field, which presented the ground truth, was not needed during training. The network was designed by cascading three full‐resolution residual subnetworks with different architectures. The training loss consisted of the image similarity loss and the deformation vector field regularization loss, which were calculated based on the final warped image and the fixed image, allowing all cascades to be trained jointly and perform the progressive registration cooperatively. Extensive network testing was conducted using diverse datasets, including an internal 4D‐CT dataset, a public DIRLAB 4D‐CT dataset, and a 4D cone‐beam CT (4D‐CBCT) dataset. Compared with the iteration‐based demon method and two deep learning‐based methods (VoxelMorph and recursive cascaded network), the RCFRR‐Net achieved consistent and significant gains, which demonstrated that the proposed method had superior performance and generalization capability in medical image registration. The proposed RCFRR‐Net was a promising tool for various clinical applications.

**Translational Impact Statement**

This study proposed a novel recursive cascaded full‐resolution residual network (RCFRR‐Net) for abdominal four‐dimensional computed tomography (4D‐CT) image registration. The proposed method showed superior performance compared with the iteration‐based demon method and two popular deep learning‐based methods (VoxelMorph and recursive cascaded network). With outstanding performance and the general applicability of the unsupervised manner, the authors expected that the proposed architecture could potentially be extended to all deformable image registration tasks.

## 1 INTRODUCTION

Four‐dimensional computed tomography (4D‐CT) was referred to as a technique whereby the three‐dimensional computed tomography (3D‐CT) volume containing a moving structure was imaged over a period, creating a dynamic volume dataset.^1^ 4D‐CT had previously been widely utilized in radiation oncology for planning purposes, especially for tumors located in the thoracic cavity, abdomen, and breast. In recent years, 4D‐CT has opened avenues in the diagnostic arena.^2^ In the diagnosis of central nervous system diseases, 4D‐CT could be utilized to evaluate vascular pathology in the brain and spine. Additionally, 4D‐CT provided the added benefits of perfusion maps and the visualization of structural changes, such as hydrocephalus or hematoma, which could aid in surgical planning. It was superior to current time‐resolved MR angiography in terms of both spatial and temporal resolution.^3^ For cardiac imaging, 4D‐CT could be used to visualize the heart and pulmonary vessels and further characterize and measure the in‐vivo 3D deformations of both the right ventricular outflow tract and the pulmonary arteries throughout the cardiac cycle.^4^

Deformable image registration (DIR) was essential in the clinical application of 4D‐CT. DIR was the process of establishing the non‐linear correspondences between an image pair of moving and fixed images. Though DIR had been extensively studied for decades, it remained an active area of research since its current performance did not fully meet the increasingly high accuracy and speed requirements for registration. Furthermore, the large and complex lung motions and low image contrast of abdominal 4D‐CT images posed additional difficulties for accurate registration.

The current DIR methods could be divided into two categories: (1) the non‐deep learning (DL)‐based methods; and (2) the DL‐based methods. Popular non‐DL‐based registration methods included two categories: the deformation vector field (DVF) optimization‐based methods and the diffeomorphic transform‐based methods. The DVF optimization‐based methods included elastic matching,^5^, ^6^ statistical parametric mapping,^7^ Demons,^8^, ^9^ etc. Popular diffeomorphic transform‐based methods included large diffeomorphic distance metric mapping (LDDMM),^10^, ^11^ diffeomorphic anatomical registration through exponentiated lie algebra (DARTEL),^12^ standard symmetric normalization (SyN).^13^ All these non‐DL‐based approaches optimized the transformation for each image pair, resulting in slow registration, especially for 3D and 4D images.

The DL‐based DIR methods have been reported to have superior performance in brain MR images, head/neck CT images, chest CT images, lung 4D‐CT images, and more.^14^, ^15^, ^16^, ^17^ According to the output of the network, the deep learning‐based DIR methods could be divided into two categories: (1) DL‐based similarity calculation; and (2) DL‐based DVF prediction. The DL‐based similarity calculation method was normally developed for multi‐modality image registration. The similarity was normally used by incorporating traditional registration methods for iterative optimization or another DL network. For example, Cheng et al.^18^ proposed a DL‐based similarity method that trained a binary classifier to learn the correspondence of two image patches from CT and MR image pairs. The classification output was transformed into a continuous probability value, which was then used as the similarity score.

The DL‐based DVF prediction method included supervised and unsupervised strategies. For the registration methods based on the supervised learning strategy, the true DVF (i.e., ground truth) calculated based on the moving image and fixed image was used to train the registration network. Three methods were normally used to generate the ground truth: (1) random DVF generation; (2) traditional registration method‐based DVF generation; and (3) DVF simulation generation. For the random DVF generation method, the generated DVF was used as the ground truth. The original image (the fixed image) was warped based on the simulated DVF to generate the moving image. However, the simulated DVF might introduce bias during training since the simulated DVF was unrealistic and quite different from actual physiological motion. For the traditional registration method‐based DVF generation method, the traditional registration methods were used to register the image pair to generate the ground truth for registration network training. Using this method, the performance of the developed registration network was greatly affected by the DVF generation methods. Further, finite element (FE) simulations could also be used in DVF generation.^19^

Compared with the supervised learning strategy‐based registration methods, the unsupervised learning strategy‐based registration methods only required the image pairs to be registered without the true DVF during model training. In general, the unsupervised learning‐based registration methods calculated the DVF using the input image pair and then performed interpolation and deformation on the moving image to generate the warped image.^20^, ^21^ The unsupervised learning strategy‐based registration methods could be classified into three categories, including generative adversarial network (GAN) methods, reinforcement learning (RL) methods, and convolutional neural network (CNN) methods.

The GAN‐based methods were mostly used to register the multi‐modality images by mapping images from one modality to another. Mahapatra et al.^22^ trained a GAN to perform registration between retinal color fundus images and fluorescein angiography images. Tanner et al.^23^ employed CycleGAN for DIR between MR and CT images. They firstly transformed the source domain to the target domain before calculating a monomodality image similarity. They discovered this method could achieve, at best, similar performance to the traditional multi‐modality deformable registration methods. Further, the accuracy of the absolute intensity mapping of GAN remained to be investigated. The combination of RL and CNN had been applied to decompose the registration task into a sequence of classification problems. This method was mainly employed for rigid registration. Liao et al.^24^ used RL to perform rigid cone‐beam CT (CBCT)‐CT image registration. Krebs et al.^25^ built a statistical deformation model (SDM) for prostate 2D and 3D MR image registration. The method achieved dice similarity coefficient scores of 0.87 and 0.80 for 2D and 3D images, respectively.

The unsupervised learning‐based CNN methods presented comparable performance to the traditional registration methods recently.^21^, ^26^, ^27^ The proposition of spatial transformation networks (STN) promoted the development of unsupervised deep learning‐based image registration methods since the STN allowed the definition of loss functions without any manually aligned or pre‐aligned image pairs.^28^ de Vos et al.^21^ applied the proposed STN to the field of image registration based on unsupervised learning for the first time. In this literature, the STN was directly connected to the network, and the generated DVF was used to deform the moving image. During the training process, the deformed and fixed images were used to calculate the training loss, which was back‐propagated through the differentiable warping operation and gradually optimized to minimize the value. Balakrishnan et al.^27^ presented a fast learning‐based registration framework (called VoxelMorph) for medical image registration. In their study, the registration task was formulated as a function that mapped the input image pair to a DVF that aligned the input images. The function was parameterized via the CNN and optimized based on the input image pairs. The studies of de Vos et al. and Balakrishnan et al. both indicated that the proposed unsupervised methods outperformed state‐of‐the‐art iterative registration methods, while operating a few orders of magnitude faster.^21^, ^27^ These proposed networks were enforced to make straightforward predictions, which might be a burden when handling complicated deformations, especially with large deformations.^29^

de Voset et al.^30^ proposed a DL image registration (DLIR) framework for unsupervised affine and deformable image registration. DLIR stacked multiple networks into a larger architecture to perform coarse‐to‐fine image registration. DLIR was trained on each cascade one by one, that was, after fixing the weights of previous cascades. The volume tweening network (VTN) was an end‐to‐end cascading scheme that resolved large displacements.^31^ VTN jointly trained all cascades, while the similarity was measured based on the fixed image and all warped images. Zhao et al.^29^ presented a general recursive cascaded networks (RC‐Net) architecture that enabled learning deep cascades for deformable image registration. The registration procedure was recursive, such that every cascade learned to perform a progressive deformation for the current warped image. The similarity was only measured based on the fixed image and the final warped image. The base network of the RC‐Net had a simple and identical architecture of VoxelMorph and VTN. In addition, the base network had the same network depth and parameters. One drawback of the RC‐Net was that the number of parameters increased linearly with the number of stacks. The RC‐Net with five cascades showed a Dice value of 0.949. The RC‐Net achieved a Dice value of 0.953 to 0.956 from 10 to 20 cascades using the VTN base network. With a greater number of cascades, the improvement in registration performance might be limited.

This article made several technical contributions towards the goal of developing neural networks for abdominal 4D‐CT image registration. (i) We introduced a novel registration network with three recursive cascaded subnetworks to perform progressive registration. Each subnetwork was designed with different architectures to capture different movement conditions. (ii) We proposed to calculate the DVF based on the full‐resolution residual network, which could capture the precise boundary and texture information of the image simultaneously. (iii) We evaluated the proposed network using multiple datasets (an internal 4D‐CT dataset, a public DIRLAB 4D‐CT dataset, and a 4D‐CBCT dataset).

## 2 MATERIALS AND METHODS

### 2.1 Workflow

**FIGURE 1:**
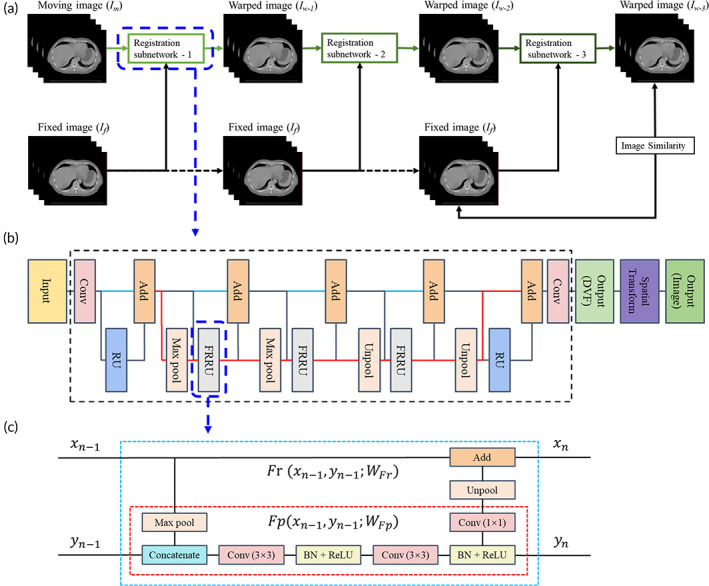
a: Illustration of the proposed network architecture. The network included three cascade registration subnetworks. The moving image was recursively and progressively warped by each cascade, finally aligned to the fixed image. b: Example of the registration subnetwork. Each cascaded subnetwork consisted of a DVF prediction network and a spatial transformation function. The layers within the black dotted box indicated the DVF prediction network. c: Illustration of the structure of the a full‐resolution residual unit (FRRU). The red dotted box indicated the function of $\mathrm{Fp}\left(x_{n-1},y_{n-1};W_{\mathrm{Fp}}\right)$. The blue dotted box indicated the function of $\mathrm{Fr}\left(x_{n-1},y_{n-1};W_{\mathrm{Fr}}\right)$.

Figure 1a depicted the workflow of the proposed recursive cascaded full‐resolution residual network (RCFRR‐Net). Considering the large and complex motion patterns of abdominal 4D‐CT images, the recursive cascaded method was used to conduct successive registration. The RCFRR‐Net had three independent registration subnetworks with different network parameters and depths, aiming to perform global and refined registrations. Each subnetwork was directly connected and used as an independent network with respective input  and output. The first subnetwork used the fixed and moving image pairs as inputs. The second and third subnetworks used the fixed image and the warped image (output of the front registration subnetwork) as the paired input images. To accurately capture the precise boundary information of image elements, the subnetwork was developed by merging a full‐resolution residual network (FRRN) and a spatial transformation function. The loss of the RCFRR‐Net was calculated based on the fixed image and the final warped images during the network training process. The trained network was applied to the input image data to calculate the final warped image to achieve registration.

### 2.2 Recursive cascaded networks

The fixed and moving images were defined over the 3D spatial domain. The work in this paper focused solely on the 3D image volume with grayscale data. Theoretically speaking, the proposed method was dimension‐independent. The DVF was the mapping of ϕ:Ω→Ω. For DIR, a reasonable and plausible DVF should be continuously varying. A DVF prediction function, $R_{\theta }\left(I_{f},I_{m}\right)$, was developed in the proposed method, where θ indicated the network parameters, $I_{f}$ indicated the fixed images and $I_{m}$ indicated the moving images. The function $R_{\theta }$ used the $I_{f}$, $I_{m}$ as inputs and predicted the DVF (ϕ) and the warped image $I_{w}$ as outputs. The $I_{w}$ was generated based on the predicted DVF and the moving image:

(1) $$I_{w}=I_{m}\circ \phi$$

The entire registration task was cascaded by recursively performing registration on the warped images. The $R_{\theta }$ included three cascaded registration subnetworks, denoting as $r_{1}$, $r_{2}$, and $r_{3}$. Following the recursion, the moving image was warped successively three times. Each cascaded subnetwork was an independent image registration network. The *n*‐th cascaded subnetwork ($r_{n}$) could predict the DVF ($\phi_{n}$) based on the input warped image ($I_{w}^{n-1}$) and the fixed image, as Equation (2). The predicted DVF of the entire network ($\phi_{\text{RCFRR}-\mathrm{Net}}$) was the composition of the DVFs of the three cascaded subnetworks:

(2) $$\begin{array}{c} \phi_{n}=r_{n}\left(I_{f},I_{w}^{n-1}\right) \end{array}$$

(3) $$\begin{array}{c} \phi_{\text{RCFRR}-\mathrm{Net}}=\phi_{1}\circ \phi_{2}\circ \phi_{3} \end{array}$$

The final warped image ($I_{w}^{3}$) was generated based on the warped image ($I_{w}^{2}$) of $r_{2}$ and the predicted DVF ($\phi_{3}$) of $r_{3}$, which could also be expressed using the input moving image and the final predicted DVF, as:

(4) $$\begin{array}{c} I_{w}^{3}=I_{w}^{2}\circ \phi_{3}=I_{m}\circ \phi_{\text{RCFRR}-\mathrm{Net}} \end{array}$$

### 2.3 Registration subnetworks

Each cascaded registration subnetwork consisted of a DVF prediction network and a spatial transformation function. Each subnetwork was designed to predict a DVF and a warped image of itself based on the input moving image (or warped image from the front registration subnetwork) and the fixed image. The DVF prediction network was designed to compute the DVF. The spatial transformation function was used to warp the input moving image based on the predicted DVF.

The DVF prediction network was implemented using a ResNet‐similar architecture, the full‐resolution residual network (FRRN).^32^, ^33^ An example of the abstract structure of the FRRN was provided in Figure 1b. The FRRN processed two distinct feature streams: a residual stream and a pooling stream. The residual stream carried feature maps at full resolution with precise boundary information. The features of the residual stream were calculated by adding successive residual units. The pooling stream carried high‐level feature maps with information on image texture. The features of the pooling stream were the direct output of the multiple convolution and pooling operations applied to the input images. By fusing the two processing streams, both kinds of features could be calculated simultaneously.

The FRRN was constructed using a sequence of full‐resolution residual units (FRRUs).^32^ The abstract structure of the FRRU was presented in Figure 1c. Since the FRRN could process the residual stream and pooling stream synchronously, each FRRU had two inputs and two outputs accordingly. Let $x_{n-1}$ denote the input of the residual stream to the *n*th FRRU and $y_{n-1}$ denote the input of the pooling input. The outputs, functions of the residual stream (Fr), and pooling stream (Fp) of the *n*th FRRU were defined as:

(5) $$\begin{array}{c} x_{n}=x_{n-1}+\mathrm{Fr}\left(x_{n-1},y_{n-1};W_{\mathrm{Fr}}\right) \end{array}$$

(6) $$\begin{array}{c} y_{n}=\mathrm{Fp}\left(x_{n-1},y_{n-1};W_{\mathrm{Fp}}\right) \end{array}$$

where $W_{\mathrm{Fr}}$ and $W_{\mathrm{Fp}}$ were the parameters of the residual stream and pooling stream, respectively. In Figure 1c, the red box indicated the function of the pooling stream, while the blue box indicated the function of the residual stream.

The FRRU firstly reduced the size of the feature map of the residual stream using a max‐pooling layer and then concatenated the two feature maps of the two streams. Next, the concatenated feature maps underwent two 3 × 3 convolution layers, aiming to extract highly local and complex features. Each convolutional layer was followed by a spatial batch normalization (BN) layer and a rectified linear activation function (ReLU).^33^ The outputs of the latter convolution layer were fed into the pooling stream and residual stream of the next FRRU. For the pooling stream, the outputs were directly used as inputs. For the residual stream, we adjusted the number of feature channels using a 1 × 1 convolution layer and upscaled the spatial dimensions using an unpooling layer.

To use the standard gradient‐based optimization method, the spatial transformation function‐based differentiable operation was used to calculate $I_{m}\circ \phi .$ ^28^ For the voxel v of the $I_{m}$, the voxel (subpixel) location $\phi \left(v\right)$ was calculated. Since the voxels were only defined at the integer locations, the voxel of $I_{m}\circ \phi \left(v\right)$ was calculated based on the neighboring voxels using linear interpolation:

(7) $$\begin{array}{c} I_{m}\circ \phi \left(v\right)=\sum_{w\in Z\left(\phi \left(v\right)\right)}I_{m}\left(w\right)\prod_{d\in \left\{x,y,z\right\}}\left(1-\left|\phi_{d}\left(v\right)-w_{d}\right|\right) \end{array}$$

where $Z\left(\phi \left(v\right)\right)$ indicated the voxel neighbors of $\phi \left(v\right)$. Since the spatial transformation function was differentiable, the errors could be backpropagated during the training process.

**FIGURE 2:**
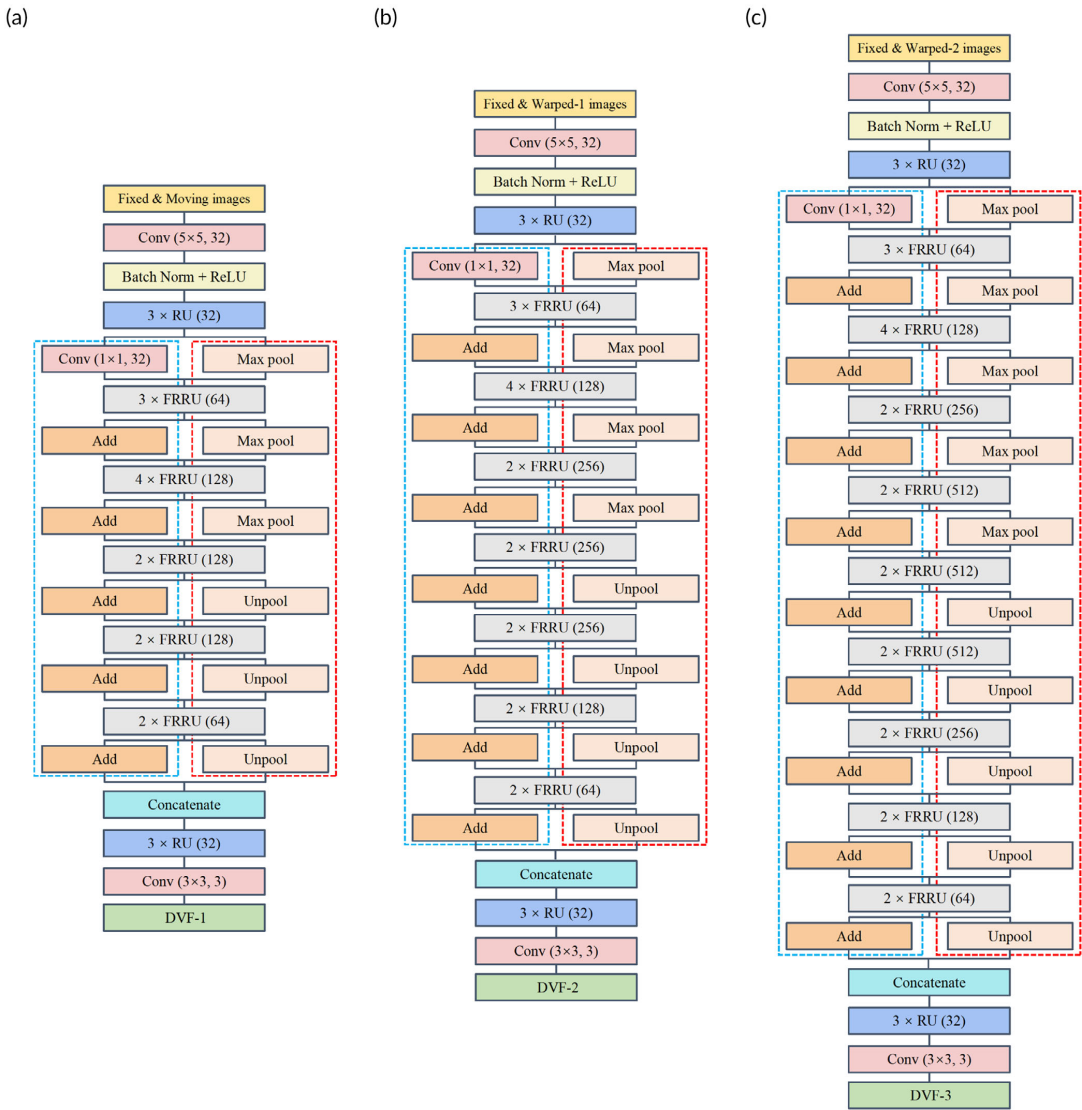
The network architectures of the three cascade registration subnetworks. The notation Conv (*n × n, m*) indicated a convolution layer of *m* kernels with each of size *n × n*. The notations RU(*v*) and FRRU(*v*) indicated the RU and FRRU whose convolutions included *v* channels. The three cascade registration subnetworks differed in network depth. (a) Subnetwork‐1; (b) Subnetwork‐2; and (c) Subnetwork‐3.

The three cascade registration subnetworks were designed with different network parameters and depths in this study. The top (first) cascade was designed to learn global registration with a shallow network architecture (FRRU blocks: 5, maximum number of FRRU channels: 128). The second cascade was designed with a deeper network architecture compared with the first cascade (FRRU blocks: 7, maximum number of FRRU channels: 256). The bottom cascade was designed to learn the refined registration with the deepest network architecture (FRRU blocks: 9, maximum number of FRRU channels: 512). The proposed architectures were constructed based on principles employed by state‐of‐the‐art methods.^32^, ^34^, ^35^ The base channel, which was different from Simonyan and He's studies, was set to 32 to have a manageable number of trainable parameters.^34^, ^36^ The channel number was doubled after each pooling operation (up to the certain maximum number). The encoder and decoder formulations were used according to Noh and Pohlen's studies.^32^, ^35^ The network architectures of the three cascaded registration subnetworks were presented in Figure 2.

### 2.4 Loss function

Three cascaded registration subnetworks were jointly trained by merely calculating the unsupervised loss ($L_{\mathrm{us}}$) between the fixed image and the final warped image.^29^ This allowed the recursive cascaded networks to learn to perform progressive registration cooperatively. The loss function consisted of two components, which were the image similarity loss ($L_{\mathrm{sim}}\left(I_{f},I_{w}\right)$) and the DVF regularization loss ($L_{\text{smooth}}\left(\phi \right)$).^27^ The image similarity loss penalized the differences in image appearance between $I_{f}$ and $I_{w}^{3}$. The DVF regularization loss penalized the local spatial variations in DVF.

(8) $$\begin{array}{c} L_{\mathrm{us}}\left(I_{f},I_{w},\phi \right)=\min_{L_{\mathrm{us}}}\left\{L_{\mathrm{sim}}\left(I_{f},I_{w}^{3}\right)+\alpha L_{\text{smooth}}\left(\phi_{3}\right)\right\} \end{array}$$

where α indicated the regularization parameter. The α was set as 0.01 according to the previous work of Balakrishnan et al.^27^

The $L_{\mathrm{sim}}\left(I_{f},I_{w}^{3}\right)$ was conducted using the mean squared error:

(9) $$\begin{array}{c} L_{\mathrm{sim}}\left(I_{f},I_{w}^{3}\right)=\frac{1}{\left|\Omega \right|}\sum_{p\in \Omega }{\left[f\left(p\right)-w\left(p\right)\right]}^{2} \end{array}$$

Minimizing the term $L_{\mathrm{sim}}\left(I_{f},I_{w}^{3}\right)$, would enforce $I_{w}^{3}$ to approximate $I_{f}$. It might generate an unrealistic non‐smooth DVF. The DVF regularization loss encouraged a smooth DVF using the diffusion regularizer on the spatial gradients:

(10) $$\begin{array}{c} L_{\text{smooth}}\left(\phi_{3}\right)=\sum {\left\|\nabla \phi_{3}\right\|}^{2} \end{array}$$

### 2.5 Experiments

#### 2.5.1 Dataset

The proposed RCFRR‐Net was trained and tested using a retrospective dataset involving 13 patients' 4D‐CT images. All images were obtained from Siemens SOMATOM Definition AS using 120 kVp tube voltages. The original image pixels of the datasets ranged from 0.6836 to 0.9736 mm. The slice thickness ranged from 2 to 3 mm. All images were cropped and resampled to the same size of 96 × 96 × 96. Image gray values were normalized to [0, 1]. Each 4D‐CT dataset included 11 phases of 3D‐CT images throughout the respiratory cycle: one initial phase (denoted as: In0%), five exhalation phases (denoted as: Ex20%, Ex40%, Ex60%, Ex80%, and Ex100%), and five inhalation phases (denoted as: In20%, In40%, In60%, In80%, and In100%). The 4D‐CT datasets of 12 patients, including a total of 132 3D‐CT images, were used as the training dataset. During training, image pairs between any two phases of the 11 phases were used as the moving image and the fixed image. In total, the training dataset included 1320 pairs of 3D‐CT images. For testing, the 3D‐CT image of the exhale and inhale phases was registered to the initial phase image. A total of 10 CT image pairs were used for testing.

The performance of the proposed RCFRR‐Net was tested using the public DIRLAB dataset (www.dir-lab.com). The 3D images were resampled to the same size of 96 × 96 × 96 and normalized to the same range of 0 to 1. Each 4D‐CT of the DIRLAB dataset included 10 phases. The 3D image of the first phase was used as the fixed image, while the images of the other nine phases were used as the moving images. Nine image pairs were generated for each case. Three cases (case 1, case 2, and case 3) from DIRLAB were included. Thus, a total of 27 image pairs were used for testing based on the DIRLAB dataset.

Furthermore, to test the generalization capability of the proposed RCFRR‐Net, the authors tested the proposed RCFRR‐Net using 4D‐CBCT images. The images were obtained from “*The Sparse‐view Reconstruction Challenge for Four‐dimensional Cone‐beam CT*” (https://image-x.sydney.edu.au/spare-challenge/). The 4D‐CBCT images had higher noise and artifact levels compared with the 4D‐CT images. Each 4D‐CBCT dataset also included 10 phases. The 3D images were resampled and normalized prior to testing. The 3D image of the first phase was used as the fixed image, and the 3D images of the other nine phases were used as the moving images. A total of nine CBCT image pairs were included for the 4D‐CBCT testing.

#### 2.5.2 Evaluation

To compare the registration performance of the proposed RCFRR‐Net with other DIR methods, we used the iteration‐based demon registration method as the first comparison method.^9^ In addition, two deep learning‐based methods, VoxelMorph, and recursive cascaded networks (RC‐Net), were also included as comparison methods.^27^, ^29^ VoxelMorph and RC‐Net were trained using the same training dataset. The VoxelMorph and RC‐Net were both constructed based on the *vm2double* architecture. The hyperparameters of the two networks were consistent with the previous papers.^27^, ^29^ We developed two RC‐Nets with three and 10 cascades, abbreviated as RC‐Net‐3 and RC‐Net‐10, respectively.

**FIGURE 3:**
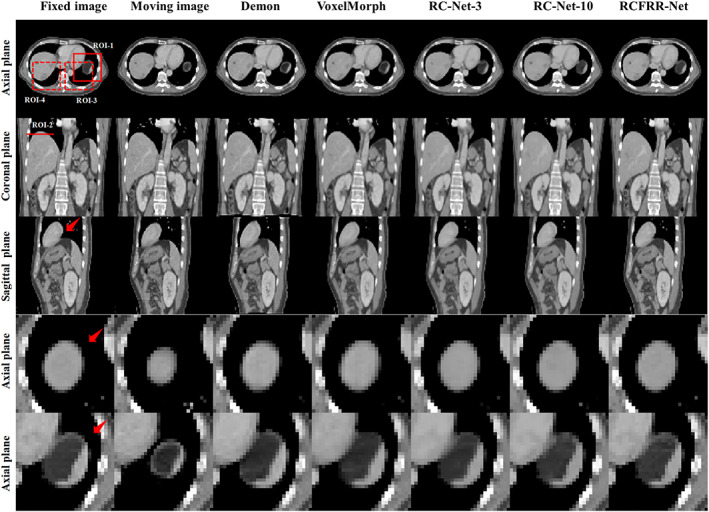
Comparison of the moving images, fixed images, and warped images of different registration methods. The first three rows showed the comparison of global images, and the last two rows showed the comparison of ROI‐1 and ROI‐2. The red box in the axial plane and the line in the coronal plane indicated the example slices of ROIs. The red arrow in the figures indicated the organs with large motion. The images of the six columns were the fixed images, moving images, and warped images of demon‐based registration, VoxelMorph, RC‐Net‐3, RC‐Net‐10, and RCFRR‐Net, respectively. Display window: [‐200, 300] HU.

Root mean square error (RMSE), normalized cross‐correlation (NCC), structural similarity index (SSIM), and Dice score were calculated for quantitative evaluations.^37^, ^38^ Considering that the organs in the abdomen, for example, the liver and stomach, had large amplitudes of motion during the respiratory process, the registration accuracy was also measured based on these key organs. Four regions of interest (ROIs), including the liver, stomach, lung, and surroundings, were selected from the global image to calculate the quantitative metrics for registration evaluation. The ROIs had the same size of 30*30*5. The ROI‐1 and ROI‐3 were chosen in the stomach and lung regions. The ROI‐2 and ROI‐4 were chosen in the liver and lung regions. The example slices of the ROIs were presented in Figure 3.

The RMSE indicated the squared differences of voxel intensities of two images and was defined as:

(11) $$\begin{array}{c} \text{RMSE}=\sqrt{\frac{1}{m}\sum_{i=1}^{m}{\left(\mu_{w}^{i}-\mu_{f}^{i}\right)}^{2}} \end{array}$$

where *i* indicated the index of the voxel in the image, m indicated the total number of the voxels in the image, $\mu_{w}^{i}$ was the intensity value of the *i*th voxel in the warped image, and $\mu_{f}^{i}$ was the intensity value of the *i*th voxel in the fixed image.

The NCC was used to measure the correlation between two images:

(12) $$\begin{array}{c} \mathrm{NCC}=\frac{\sum_{i=1}^{m}\left(\mu_{w}^{i}-\bar{\mu_{w}}\right)\left(\mu_{f}^{i}-\bar{\mu_{f}}\right)}{\sqrt{\left(\sum_{i=1}^{m}{\left(\mu_{w}^{i}-\bar{\mu_{w}}\right)}^{2}\right)\left(\sum_{i=1}^{m}{\left(\mu_{f}^{i}-\bar{\mu_{f}}\right)}^{2}\right)}} \end{array}$$

where $\bar{\mu_{w}}$ and $\bar{\mu_{f}}$ were the mean values of the warped image and fixed image, respectively.

The SSIM indicated the similarity of the two images regarding three terms, namely the intensity term, the contrast term, and the structural term. It was proposed to provide a good approximation of perceptual image quality:

(13) $$\begin{array}{c} \text{SSIM}=\frac{\left(2\bar{\mu_{w}}\bar{\mu_{f}}+C_{1}\right)\left(2\sigma_{\mathrm{wf}}+C_{2}\right)}{\left({\bar{\mu_{w}}}^{2}+{\bar{\mu_{f}}}^{2}+C_{1}\right)\left(\sigma_{w}^{2}+\sigma_{f}^{2}+C_{2}\right)} \end{array}$$

where $C_{1}={\left(0.01\times L\right)}^{2}$ and $C_{2}={\left(0.03\times L\right)}^{2}$ were the small constants to avoid instability of SSIM calculation. Here, L was the dynamic range of the input image. $\sigma_{w}$ and $\sigma_{f}$ were the standard deviations of the warped image and fixed image, respectively. $\sigma_{\mathrm{wf}}$ was the cross‐covariance between the warped and fixed images.

The Dice score was a spatial overlap index. In our study, the Dice score was calculated based on the selected ROIs. The Dice score was measured:

(14) $$\begin{array}{c} \text{Dice}=2\times \frac{{\mathrm{ROI}}_{W}\cap {\mathrm{ROI}}_{F}}{\left|{\mathrm{ROI}}_{W}\right|+\left|{\mathrm{ROI}}_{F}\right|} \end{array}$$

where ${\mathrm{ROI}}_{W}$ and ${\mathrm{ROI}}_{F}$ were the ROIs of the warped image and fixed image, respectively.

#### 2.5.3 Implementation

The proposed algorithm was implemented in Python 3.6 and PyTorch 1.7.0 framework.^39^ The proposed model was trained using a batch size of 1 on a NVIDIA GeForce RTX 3090 GPU with 24 GB of memory. The training stage ran for 9 × 10^4^ iterations with the Adam gradient optimizer.^40^ The learning rate was initially 10^−4^ and halved after 3 × 10^4^ iterations and again after 6 × 10^4^ iterations.

## 3 RESULTS

### 3.1 Registration evaluation of RCFRR‐Net

**FIGURE 4:**
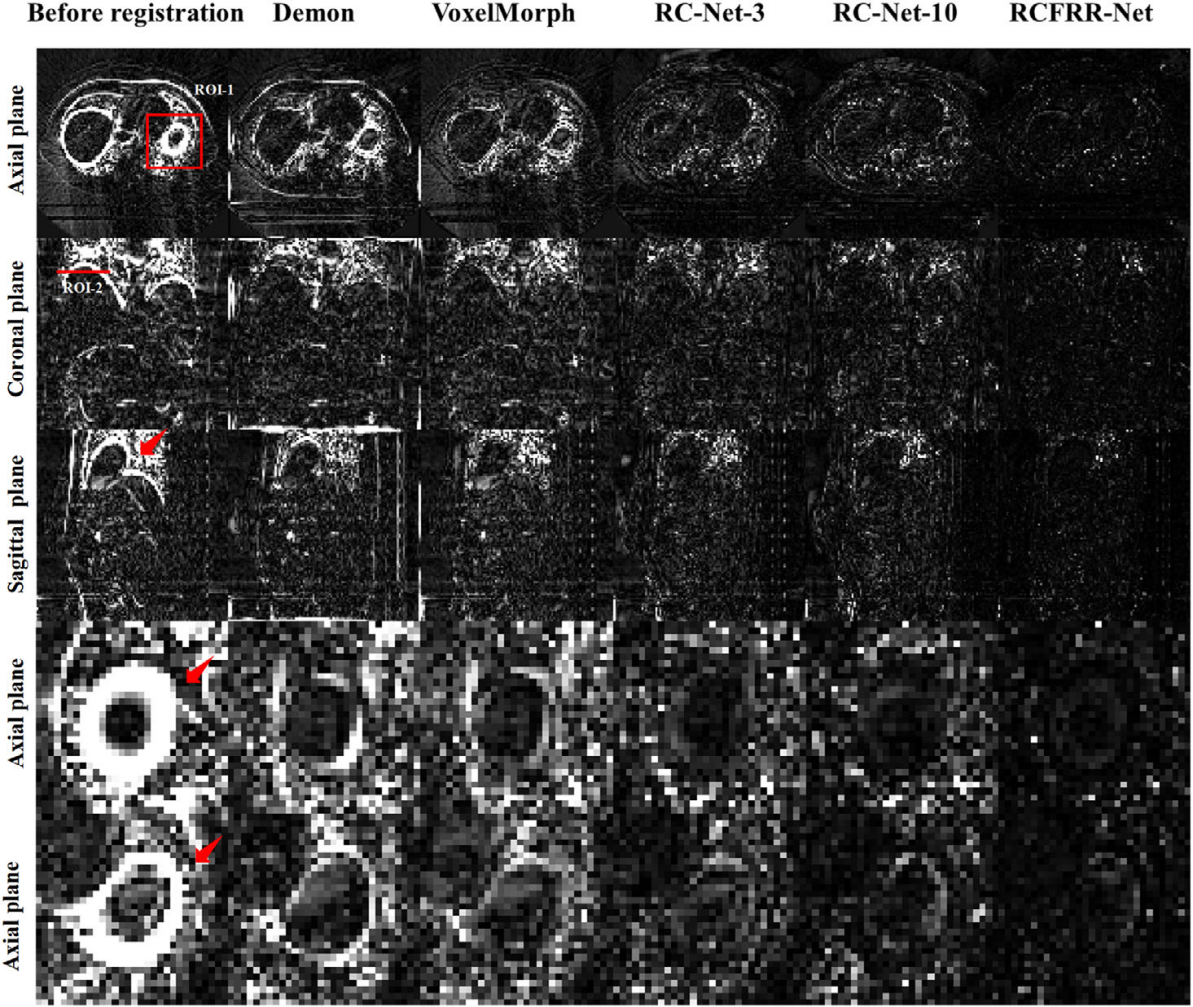
Comparison of the residual images between the fixed images and warped images of different registration methods. The first three rows showed the comparison of global images, and the last two rows showed the comparison of ROIs. The red box in the axial plane and line in the coronal plane indicated ROI‐1 and ROI‐2, respectively. The red arrow in the figures indicated the organs with large motion. The images of the five columns were the residual images between fixed images and moving images, warped images of demon‐based registration, VoxelMorph, RC‐Net‐3, RC‐Net‐10, and RCFRR‐Net, respectively. Display window: [0, 100] HU.

Table 1 showed the global image and ROI‐based comparison of the average registration results using different registration methods over the 10 image pairs of the testing patient. As seen in Table 1, the VoxelMorph performed better than the demon registration method according to the global image evaluation and performed comparably to the demon registration method according to the ROI‐based evaluation. The RC‐Net‐3 performed better than the VoxelMorph and demon registration methods. The RC‐Net‐10 had slightly better performance than the RC‐Net‐3. The RCFRR‐Net showed optimal performance among all registration methods. Figure 3 showed the axial, coronal, and sagittal planes and selected ROIs of the moving images, fixed images, and warped images registered using different methods. The absolute difference between the fixed images and the warped images of different registration methods were shown in Figure 4. It could be seen from the residual images that the difference images from the proposed RCFRR‐Net were smaller compared with the comparison methods, especially in the selected ROIs.

**TABLE 1:** Comparison of RMSE, NCC, SSIM, and dice score using different registration methods.

Comparison content	RMSE (HU)	NCC	SSIM	Dice score
Global image				
Before registration	82.52 ± 23.75	0.9831 ± 0.0084	0.6892 ± 0.0574	–
Demon registration	64.50 ± 20.18	0.9928 ± 0.0039	0.7014 ± 0.0462	–
VoxelMorph	27.93 ± 5.08	0.9981 ± 0.0006	0.7712 ± 0.0295	–
RC‐Net‐3	19.63 ± 2.59	0.9992 ± 0.0001	0.7855 ± 0.0289	–
RC‐Net‐10	18.64 ± 3.34	0.9994 ± 0.0001	0.8052 ± 0.0193	–
RCFRR‐Net	10.88 ± 3.78	0.9999 ± 0.0001	0.8762 ± 0.0258	–
ROI‐1				
Before registration	337.37 ± 71.01	0.6621 ± 0.1102	0.3504 ± 0.1171	0.5532 ± 0.0766
Demon registration	80.12 ± 10.46	0.9789 ± 0.0046	0.8248 ± 0.0309	0.7443 ± 0.0329
VoxelMorph	76.43 ± 15.63	0.9794 ± 0.0074	0.8267 ± 0.0379	0.7301 ± 0.0331
RC‐Net‐3	41.13 ± 5.31	0.9942 ± 0.0012	0.9122 ± 0.0152	0.8033 ± 0.0315
RC‐Net‐10	36.53 ± 3.89	0.9955 ± 0.0008	0.9234 ± 0.0158	0.8099 ± 0.0316
RCFRR‐Net	17.79 ± 2.85	0.9991 ± 0.0002	0.9695 ± 0.0091	0.8681 ± 0.0201
ROI‐2				
Before registration	261.36 ± 71.35	0.7937 ± 0.0795	0.4343 ± 0.1175	0.7836 ± 0.0464
Demon registration	56.76 ± 8.12	0.9867 ± 0.0032	0.8177 ± 0.0247	0.8879 ± 0.0182
VoxelMorph	57.85 ± 15.19	0.9843 ± 0.0077	0.7936 ± 0.0530	0.8696 ± 0.0369
RC‐Net‐3	30.49 ± 3.18	0.9962 ± 0.0008	0.8858 ± 0.0201	0.9231 ± 0.0138
RC‐Net‐10	26.09 ± 1.88	0.9973 ± 0.0003	0.9019 ± 0.0203	0.9323 ± 0.0138
RCFRR‐Net	14.58 ± 2.28	0.9993 ± 0.0001	0.9503 ± 0.0183	0.9579 ± 0.0111
ROI‐3				
Before registration	306.97 ± 63.03	0.7158 ± 0.0938	0.4834 ± 0.0697	0.7895 ± 0.024
Demon registration	80.09 ± 14.74	0.9778 ± 0.0067	0.8029 ± 0.0453	0.8346 ± 0.0272
VoxelMorph	72.13 ± 16.68	0.9802 ± 0.0079	0.8270 ± 0.0432	0.8409 ± 0.0168
RC‐Net‐3	42.34 ± 6.99	0.9936 ± 0.0017	0.8963 ± 0.0204	0.8731 ± 0.0143
RC‐Net‐10	36.89 ± 4.76	0.9950 ± 0.0012	0.9125 ± 0.0165	0.8736 ± 0.0156
RCFRR‐Net	18.13 ± 2.64	0.9990 ± 0.0002	0.9587 ± 0.0124	0.9121 ± 0.0137
ROI‐4				
Before registration	236.87 ± 61.79	0.7979 ± 0.0738	0.5736 ± 0.0716	0.7640 ± 0.0436
Demon registration	55.20 ± 9.13	0.9854 ± 0.0041	0.8450 ± 0.0200	0.8802 ± 0.0132
VoxelMorph	53.54 ± 13.42	0.9857 ± 0.0069	0.8478 ± 0.0374	0.8641 ± 0.0265
RC‐Net‐3	29.46 ± 3.01	0.9959 ± 0.0008	0.9075 ± 0.0181	0.9112 ± 0.0221
RC‐Net‐10	25.79 ± 1.67	0.9969 ± 0.0003	0.9099 ± 0.0188	0.9189 ± 0.0213
RCFRR‐Net	14.03 ± 2.07	0.9993 ± 0.0001	0.9568 ± 0.0100	0.9542 ± 0.0131

Using the quantitative metrics for the registration evaluation, the original average RMSE between the moving image and fixed image was 82.52 ± 23.75 HU. With the proposed RCFRR‐Net, the average RMSE between the warped image and fixed image after the registration decreased to 10.88 ± 3.78 HU. The NCC increased from 0.9831 ± 0.0084 to 0.9999 ± 0.0001, and the SSIM increased from 0.682 ± 0.0574 to 0.8762 ± 0.0258. For the evaluation based on selected ROIs of the moving image and fixed image, the RMSE was much higher than the RMSE based on the global images with 337.37 ± 71.01 HU, 261.36 ± 71.35 HU, 306.97 ± 63.03 HU, and 236.87 ± 61.79 HU for ROI‐1, ROI‐2, ROI‐3, and ROI‐4, respectively. The RMSE was decreased to 17.79 ± 2.85 HU, 14.58 ± 2.28 HU, 18.13 ± 2.64 HU, and 14.03 ± 2.07 HU for the selected four ROIs based on the warped image and fixed image after the registration using RCFRR‐Net. For the selected ROIs of the moving image and the fixed image, the ROI‐based NCC and SSIM were lower than the values based on the overall image. The NCC increased from 0.6621 ± 0.1102 to 0.9991 ± 0.0002 for the ROI‐1 and from 0.7937 ± 0.0795 to 0.9993 ± 0.0001 for the ROI‐2. The SSIM also increased from 0.3504 ± 0.1171 to 0.9695 ± 0.0091 for the ROI‐1 and from 0.4343 ± 0.1175 to 0.9503 ± 0.0183 for the ROI‐2. The Dice score increased from 0.5532 ± 0.0766 to 0.8681 ± 0.0201 for the ROI‐1 and from 0.7836 ± 0.0464 to 0.9579 ± 0.0111 for the ROI‐2.

**FIGURE 5:**
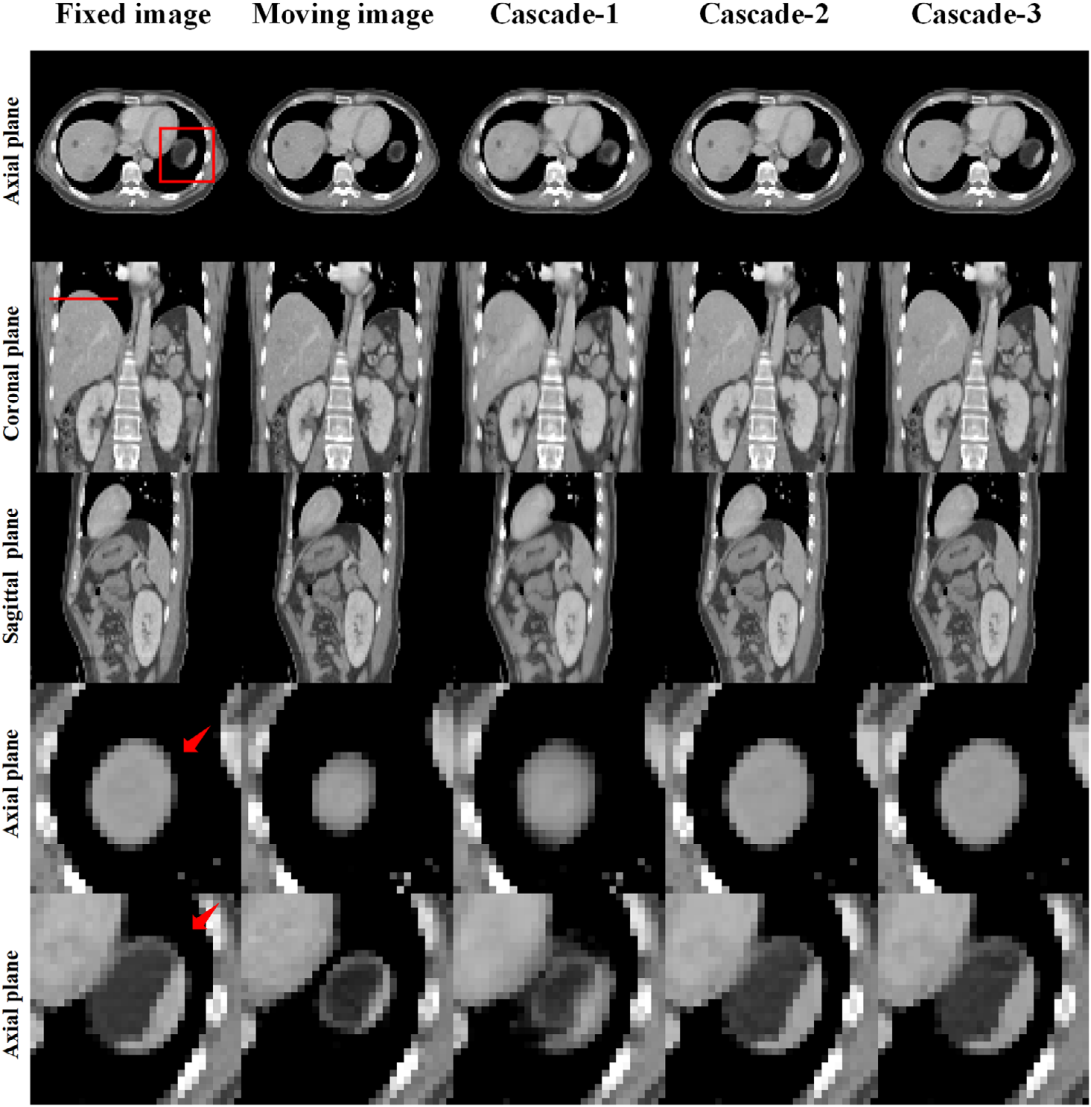
Comparison of the moving images, fixed images, and warped images of intermediate cascades. The first three rows showed the comparison of global images, and the last two rows showed the comparison of ROI‐1 and ROI‐2. The images of the five columns were the fixed images, moving images, and warped images of Cascade‐1, Cascade‐2, and Cascade‐3, respectively. Display window: [−200, 300] HU.

**FIGURE 6:**
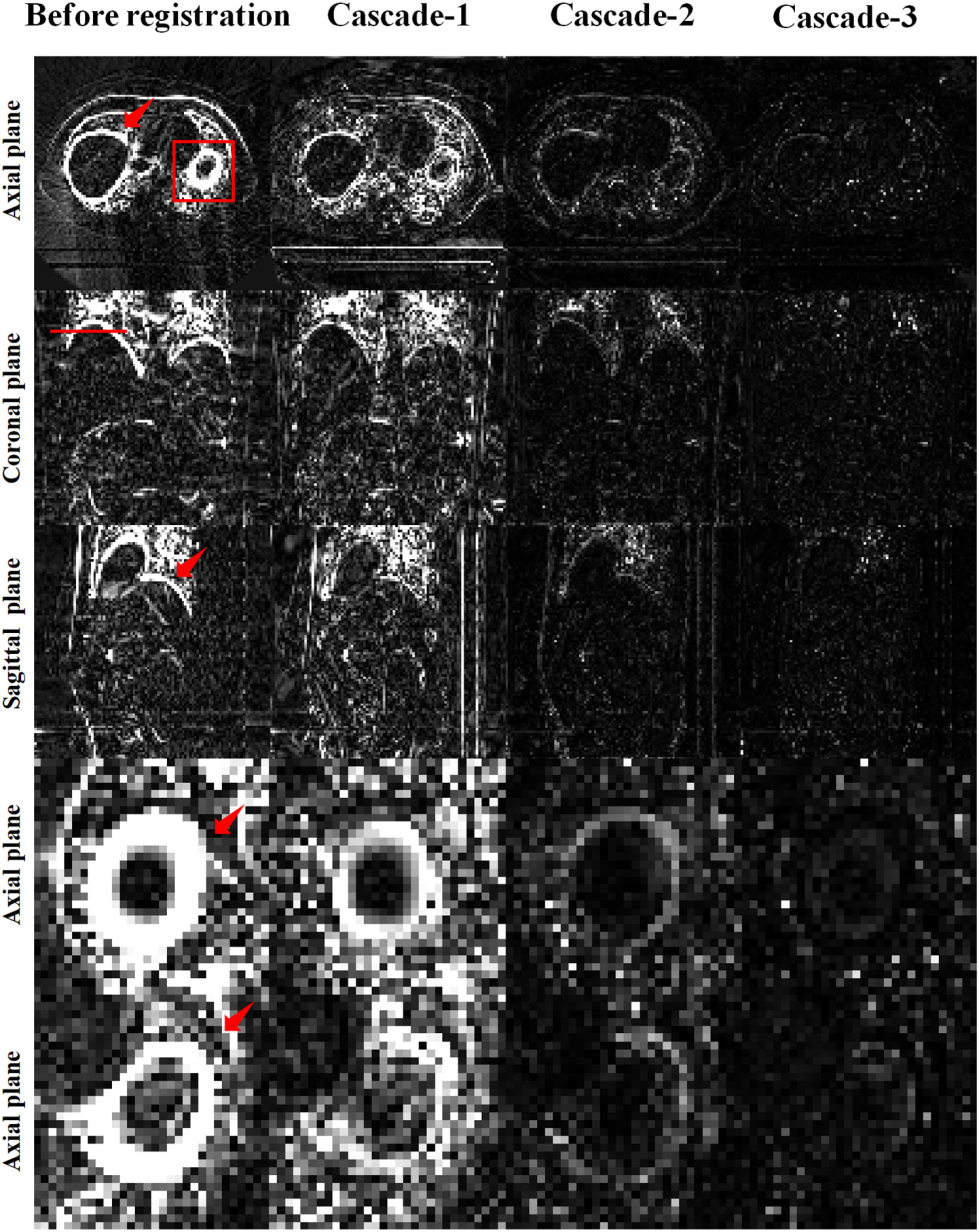
Comparison of the residual images between the fixed images and warped images of intermediate cascades. The first three rows showed the comparison of global images, and the last two rows showed the comparison of ROI‐1 and ROI‐2. The images of the four columns were the residual images between fixed images and moving images, and warped images of Cascade‐1, Cascade‐2, and Cascade‐3, respectively. Display window: [0, 100] HU.

Furthermore, to access the progressive registration process of the RCFRR‐Net, the intermediate registration results of the RCFRR‐Net were also evaluated, that was, the registration results of the first cascaded network (Cascade‐1) and the second cascaded network (Cascade‐2). Table 2 presented the results of intermediate cascades based on the global image and ROIs. As shown in the table, the RCFRR‐Net achieved performance gains with the higher cascade number. Figure 5 showed the axial, coronal, and sagittal planes and selected ROIs of the moving images, fixed images, and warped images of intermediate cascades. The absolute difference images between the fixed images and the warped images of intermediate cascades were shown in Figure 6. On average, the RMSE gradually decreased from 82.52 ± 23.75 (before registration) to 40.81 ± 4.29 for Cascade‐1, to 14.61 ± 3.50 for Cascade‐2, and finally to 10.88 ± 3.78 for Cascade‐3. The NCC and SSIM gradually increased with a higher cascade number.

**TABLE 2:** Comparison of RMSE, NCC, SSIM, and Dice score of intermediate cascades.

Comparison content	RMSE (HU)	NCC	SSIM	Dice score
Global image				
Before registration	82.52 ± 23.75	0.9831 ± 0.0084	0.6892 ± 0.0574	–
Cascade‐1	40.81 ± 4.29	0.9963 ± 0.0008	0.7014 ± 0.02009	–
Cascade‐2	14.61 ± 3.50	0.9997 ± 0.0001	0.8469 ± 0.0271	–
Cascade‐3	10.88 ± 3.78	0.9999 ± 0.0001	0.8762 ± 0.0258	–
ROI‐1				
Before registration	337.37 ± 71.01	0.6621 ± 0.1102	0.3504 ± 0.1171	0.5532 ± 0.0766
Cascade‐1	114.06 ± 27.45	0.9544 ± 0.0185	0.7006 ± 0.0735	0.6516 ± 0.0246
Cascade‐2	30.50 ± 4.02	0.9970 ± 0.0007	0.9510 ± 0.0076	0.8304 ± 0.0111
Cascade‐3	17.79 ± 2.85	0.9991 ± 0.0002	0.9695 ± 0.0091	0.8681 ± 0.0200
ROI‐2				
Before registration	261.36 ± 71.35	0.7937 ± 0.0795	0.4343 ± 0.1175	0.7836 ± 0.0464
Cascade‐1	83.91 ± 17.32	0.9692 ± 0.0109	0.7262 ± 0.0493	0.8678 ± 0.0107
Cascade‐2	24.18 ± 2.31	0.9979 ± 0.0004	0.9354 ± 0.0172	0.933 ± 0.0049
Cascade‐3	14.58 ± 2.28	0.9993 ± 0.0001	0.9503 ± 0.0183	0.9579 ± 0.0111
ROI‐3				
Before registration	306.97 ± 63.03	0.7158 ± 0.0938	0.4834 ± 0.0697	0.7895 ± 0.024
Cascade‐1	106.14 ± 24.48	0.9571 ± 0.0171	0.7036 ± 0.0653	0.7806 ± 0.0136
Cascade‐2	29.65 ± 4.02	0.9970 ± 0.0007	0.9401 ± 0.0126	0.8916 ± 0.0062
Cascade‐3	18.13 ± 2.64	0.9990 ± 0.0002	0.9587 ± 0.0124	0.9121 ± 0.0137
ROI‐4				
Before registration	236.87 ± 61.79	0.7979 ± 0.0738	0.5736 ± 0.0716	0.7640 ± 0.0436
Cascade‐1	81.11 ± 16.37	0.9664 ± 0.0121	0.7649 ± 0.0416	0.8132 ± 0.0236
Cascade‐2	22.77 ± 2.16	0.9979 ± 0.0005	0.9143 ± 0.0263	0.9214 ± 0.0064
Cascade‐3	14.03 ± 2.07	0.9993 ± 0.0001	0.9568 ± 0.0100	0.9542 ± 0.0131

**FIGURE 7:**
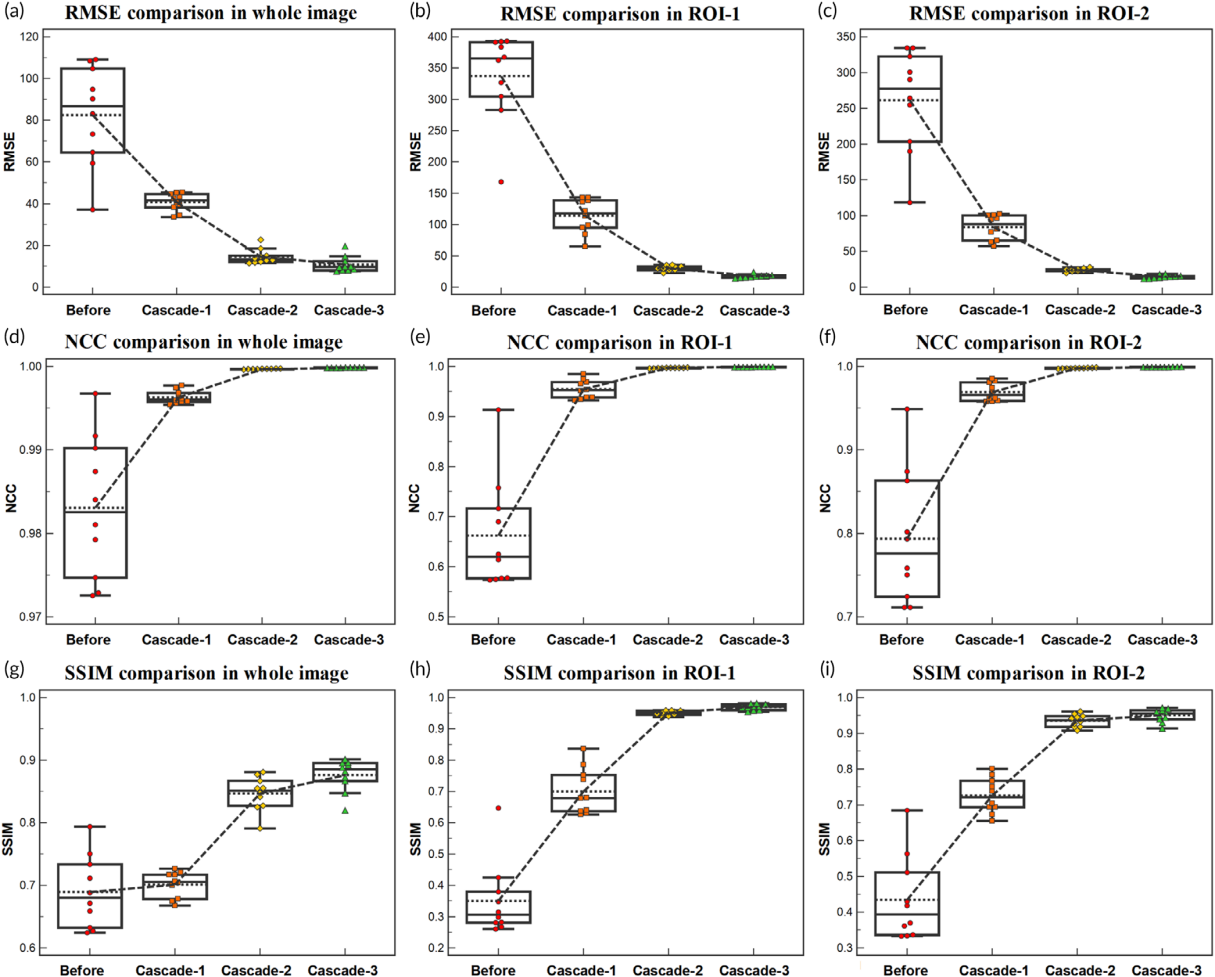
Box plots for the results of intermediate cascades. RMSE (a, b, c), NCC (d, e, f) and SSIM (g, h, i) comparison of intermediate cascades based on the global image, ROI‐1, and ROI‐2.

For the ROI‐based evaluation, the RMSE decreased from 337.37 ± 71.01 (before registration) to 114.06 ± 27.45, 30.50 ± 4.02, and 17.79 ± 2.85 for the ROI‐1 based on Cascade‐1, Cascade‐2, and Cascade‐3, respectively. The RMSE decreased from 261.36 ± 71.35 (before registration) to 83.91 ± 17.32, 24.18 ± 2.31, and 14.58 ± 2.28 for the ROI‐2 based on Cascade‐1, Cascade‐2, and Cascade‐3, respectively. The NCC, SSIM, and Dice score all gradually increased with higher cascade numbers for both ROIs. Figure 7 plotted the results of intermediate cascades to illustrate progressive registration.

### 3.2 Registration evaluation of RCFRR‐Net with different number of cascades

To measure the registration results of RCFRR‐Net with different number of cascades, we constructed RCFRR‐Net with two cascaded registration subnetworks and one registration subnetwork. The maximal number of cascades was three in the current study to consider the trade‐offs between registration time and accuracy. In this section, the RCFRR‐Net with three cascades, two cascades, and one subnetwork were denoted as RCFRR‐Net‐3, RCFRR‐Net‐2, and RCFRR‐Net‐1, respectively. The network architecture and hyper‐parameters of the RCFRR‐Net‐2 were consistent with the second and third subnetworks of the RCFRR‐Net‐3. The network architecture and hyper‐parameters of the RCFRR‐Net‐1 were consistent with the third subnetwork of the RCFRR‐Net‐3.

Table 3 presented the global image and ROI‐based comparison of the RMSE, NCC, and SSIM of RCFRR‐Net with different number of cascades. As shown in the table, RCFRR‐Net‐3 outperformed RCFRR‐Net‐1 and RCFRR‐Net‐2 as expected. For the global image‐based comparison, the RMSE was 82.52 ± 23.75 HU before registration. It was 21.90 ± 3.77, 12.86 ± 1.56, and 10.88 ± 3.78 for the RCFRR‐Net‐1, RCFRR‐Net‐2, and RCFRR‐Net‐3, respectively. The RMSE difference was 60.62 HU between the images before registration and RCFRR‐Net‐1, 9.04 HU between RCFRR‐Net‐1 and RCFRR‐Net‐2, and 1.98 HU between RCFRR‐Net‐2 and RCFRR‐Net‐3. The percentage between the RMSE difference and the RMSE before registration was 73.46%, 10.95%, and 2.4%, respectively. The RMSE difference and percentage decreased significantly with the increase in the number of cascaded networks. This trend could also be observed in the comparison based on ROIs.

**TABLE 3:** Comparison of the RMSE, NCC, SSIM, and Dice score using RCFRR‐Net with different number of cascades.

Comparison content	RMSE (HU)	RMSE Difference (HU)	NCC	SSIM
Global image				
Before registration	82.52 ± 23.75	–	0.9831 ± 0.0084	0.6892 ± 0.0574
RCFRR‐Net‐1	21.90 ± 3.77	60.62 (73.46%)	0.9989 ± 0.0004	0.7954 ± 0.0195
RCFRR‐Net‐2	12.86 ± 1.56	9.04 (10.95%)	0.9996 ± 0.0001	0.8346 ± 0.0165
RCFRR‐Net‐3	10.88 ± 3.78	1.98 (2.4%)	0.9999 ± 0.0001	0.8762 ± 0.0258
ROI‐1				
Before registration	337.37 ± 71.01	–	0.6621 ± 0.1102	0.3504 ± 0.1171
RCFRR‐Net‐1	58.78 ± 10.42	278.59 (82.58%)	0.9884 ± 0.0033	0.8753 ± 0.0228
RCFRR‐Net‐2	32.38 ± 6.52	26.4 (7.83%)	0.9962 ± 0.0015	0.9364 ± 0.0117
RCFRR‐Net‐3	17.79 ± 2.85	14.59 (4.32%)	0.9991 ± 0.0002	0.9695 ± 0.0091
ROI‐2				
Before registration	261.36 ± 71.35	–	0.7937 ± 0.0795	0.4343 ± 0.1175
RCFRR‐Net‐1	44.33 ± 10.52	217.03 (83.04%)	0.9915 ± 0.0034	0.8463 ± 0.0331
RCFRR‐Net‐2	21.72 ± 3.18	22.61 (8.65%)	0.9979 ± 0.0006	0.9273 ± 0.0110
RCFRR‐Net‐3	14.58 ± 2.28	7.14 (2.73%)	0.9993 ± 0.0001	0.9503 ± 0.0183
ROI‐3				
Before registration	306.97 ± 63.03	–	0.7158 ± 0.0938	0.4834 ± 0.0697
RCFRR‐Net‐1	79.63 ± 15.76	227.34 (74.06%)	0.9791 ± 0.0071	0.8059 ± 0.0426
RCFRR‐Net‐2	32.91 ± 7.76	46.72 (15.22%)	0.9958 ± 0.0018	0.9273 ± 0.0155
RCFRR‐Net‐3	18.13 ± 2.64	14.78 (4.81%)	0.9990 ± 0.0002	0.9587 ± 0.0124
ROI‐4				
Before registration	236.87 ± 61.79	–	0.7979 ± 0.0738	0.5736 ± 0.0716
RCFRR‐Net‐1	64.40 ± 15.49	172.27 (72.73%)	0.9800 ± 0.0079	0.8115 ± 0.0456
RCFRR‐Net‐2	20.29 ± 2.65	44.11 (18.62%)	0.9979 ± 0.0006	0.9437 ± 0.0089
RCFRR‐Net‐3	14.03 ± 2.07	6.26 (2.64%)	0.9993 ± 0.0001	0.9568 ± 0.0100

*Note*: The RMSE difference for RCFRR‐Net‐1 was calculated between the images before registration and RCFRR‐Net‐1. The RMSE difference for RCFRR‐Net‐2 was calculated between the images of RCFRR‐Net‐1 and RCFRR‐Net‐2. The percentage was calculated between the RMSE difference and RMSE before registration.

### 3.3 Registration runtime

Table 4 showed the runtime results of VoxelMorph, RC‐Net‐3, RC‐Net‐10, and RCFRR‐Net. Since the demon registration method required a few minutes to finish the registration, we only reported the runtimes of the DL‐based methods in this study. All methods were conducted using the GPU. The VoxelMorph computed a registration with an average runtime of 1.334 ± 0.0219 s. For the VoxelMorph method, the reported runtime was 0.45 s, while it had an average runtime of 1.334 ± 0.0219 s in this study.^27^ This might result from the difference in the datasets. The dataset for this study was abdominal 4D‐CT images, and the previous study was developed for brain MR datasets. Compared with the brain dataset, 4D‐CT images had more complex deformations. The runtime of the RC‐Net‐3 was slightly longer (1.446 ± 0.0207 s) than that of the VoxelMorph. The RC‐Net‐10 (1.878 ± 0.0216 s) had a longer runtime than the RC‐Net‐3. Since the subnetworks of the RCFRR‐Net were relatively deeper than the RC‐Net, the runtime of the RCFRR‐Net‐3 was slightly longer than the RC‐Net‐10 by 1.940 ± 0.0158 s. The runtimes of the RCFRR‐Net‐1, RCFRR‐Net‐2, and RCFRR‐Net‐3 increased gradually.

**TABLE 4:** Comparison of runtimes for VoxelMorph, RC‐Net, and RCFRR‐Net.

Method	GPU seconds	Range
VoxelMorph	1.334 ± 0.0219	1.31, 1.37
RC‐Net‐3	1.446 ± 0.0207	1.42, 1.47
RC‐Net‐10	1.878 ± 0.0216	1.86. 1.91
RCFRR‐Net‐1	1.572 ± 0.0356	1.54, 1.63
RCFRR‐Net‐2	1.766 ± 0.0336	1.73, 1.81
RCFRR‐Net‐3	1.940 ± 0.0158	1.92, 1.96

*Note*: The times in the PGU seconds column were presented as mean ± SD.

### 3.4 Registration evaluation on DIRLAB datasets

**FIGURE 8:**
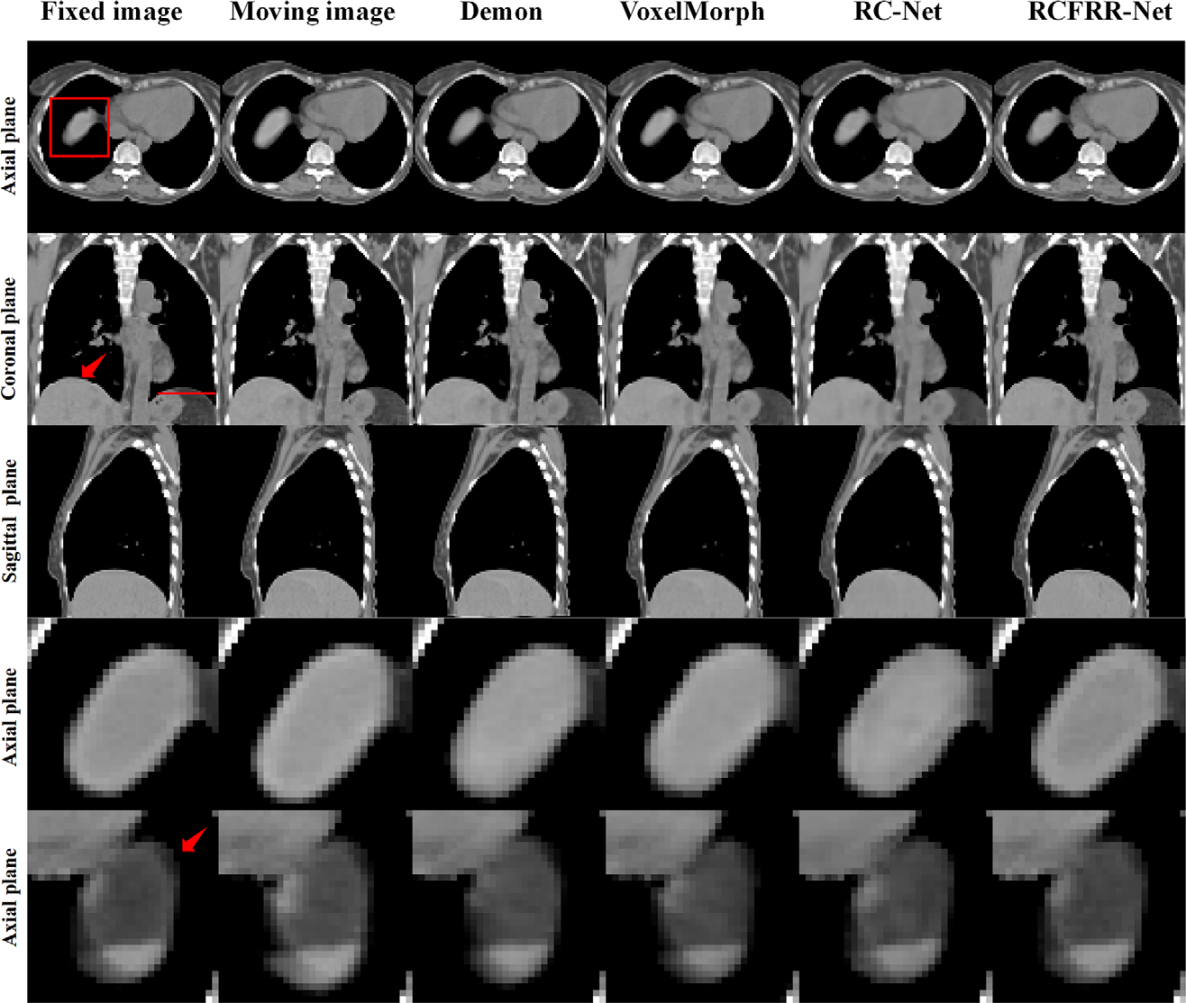
Comparison of the moving images, fixed images, and warped images of different registration methods on DIRLAB datasets. The first three rows showed the comparison of global images, and the last two rows showed the comparison of ROI‐1 and ROI‐2. The images of the six columns were the fixed images, moving images, and warped images of demon‐based registration, VoxelMorph, RC‐Net, and RCFRR‐Net, respectively. Display window: [−200, 300] HU.

**FIGURE 9:**
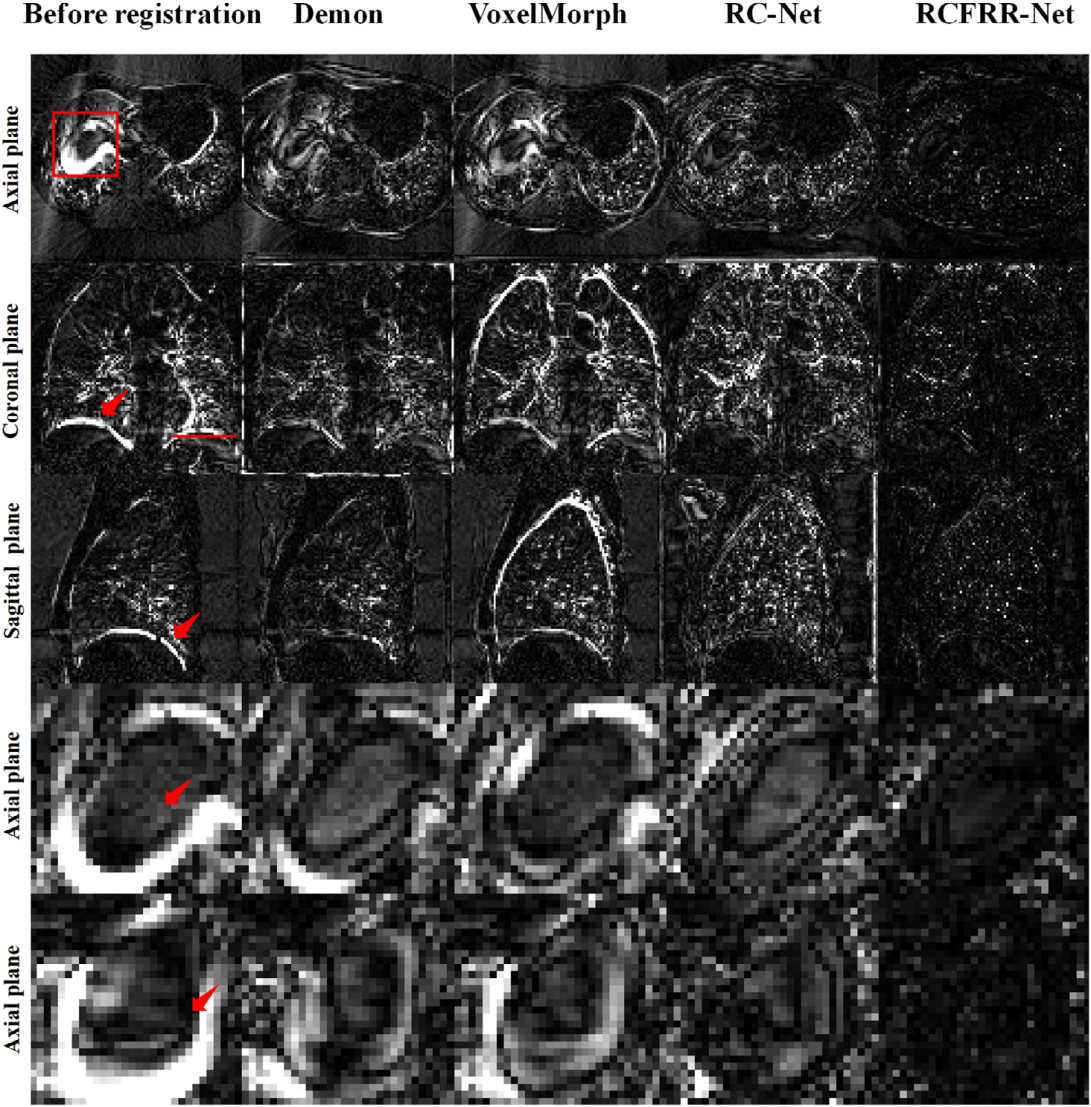
Comparison of the residual images between the fixed images and warped images of different registration methods on DIRLAB datasets. The first three rows showed the comparison of global images, and the last two rows showed the comparison of ROI‐1 and ROI‐2. The images of the five columns were the residual images between fixed images and moving images, warped images of demon‐based registration, VoxelMorph, RC‐Net, and RCFRR‐Net, respectively. Display window: [0, 100] HU.

Table 5 showed the global image and the ROI‐based comparison of the average RMSE, NCC, SSIM, and Dice score using different registration methods for the testing patients from DIRLAB datasets. Consistent with the testing dataset from our institution, the RCFRR‐Net showed optimal performance among all registration methods. Figure 8 presented the axial, coronal, and sagittal planes and selected ROIs of the moving images, fixed images, and warped images registered using different methods using images from DIRLAB datasets. The absolute difference between the fixed images and the warped images of different registration methods were shown in Figure 9. As shown in the residual images, the RCFRR‐Net showed the smallest difference.

**TABLE 5:** Comparison of RMSE, NCC, SSIM, and dice score using different registration methods based on DIRLAB datasets.

Comparison content	RMSE (HU)	NCC	SSIM	Dice score
Global image				
Before registration	78.66 ± 13.04	0.9835 ± 0.0050	0.7326 ± 0.0430	–
Demon registration	58.34 ± 12.02	0.9912 ± 0.0033	0.7649 ± 0.0379	–
VoxelMorph	45.36 ± 6.76	0.9948 ± 0.0015	0.7610 ± 0.0311	–
RC‐Net	31.53 ± 1.87	0.9977 ± 0.0003	0.7589 ± 0.0338	–
RCFRR‐Net	15.54 ± 2.47	0.9997 ± 0.0001	0.8427 ± 0.0460	–
ROI‐1				
Before registration	336.00 ± 40.13	0.6731 ± 0.0813	0.2106 ± 0.1121	0.5903 ± 0.0242
Demon registration	68.29 ± 13.50	0.9773 ± 0.0037	0.6531 ± 0.1305	0.8741 ± 0.0484
VoxelMorph	115.48 ± 22.90	0.9377 ± 0.0144	0.5457 ± 0.1082	0.7859 ± 0.0488
RC‐Net	50.57 ± 3.94	0.9881 ± 0.0024	0.6618 ± 0.0746	0.8659 ± 0.0732
RCFRR‐Net	20.12 ± 1.31	0.9983 ± 0.0006	0.8508 ± 0.0613	0.9297 ± 0.038
ROI‐2				
Before registration	339.65 ± 37.38	0.5964 ± 0.0633	0.2319 ± 0.0488	0.5497 ± 0.086
Demon registration	54.82 ± 5.97	0.9808 ± 0.0136	0.7214 ± 0.0291	0.8112 ± 0.024
VoxelMorph	101.02 ± 17.66	0.9418 ± 0.0345	0.5373 ± 0.0464	0.6261 ± 0.0406
RC‐Net	48.14 ± 4.96	0.9856 ± 0.0081	0.7010 ± 0.0200	0.7891 ± 0.0257
RCFRR‐Net	21.66 ± 0.69	0.9975 ± 0.0020	0.8978 ± 0.0074	0.9184 ± 0.0177
ROI‐3				
Before registration	197.99 ± 18.71	0.809 ± 0.0216	0.4554 ± 0.1706	0.8677 ± 0.0334
Demon registration	45.30 ± 2.85	0.9905 ± 0.0029	0.6764 ± 0.0393	0.9454 ± 0.0094
VoxelMorph	65.49 ± 2.48	0.9796 ± 0.0046	0.62 ± 0.0841	0.9257 ± 0.0105
RC‐Net	31.61 ± 7.86	0.996 ± 0.0014	0.6498 ± 0.0104	0.9558 ± 0.0082
RCFRR‐Net	16.08 ± 1.34	0.9993 ± 0.0002	0.8196 ± 0.0159	0.9735 ± 0.0061
ROI‐4				
Before registration	353.17 ± 59.46	0.5962 ± 0.0836	0.2001 ± 0.1211	0.5839 ± 0.0701
Demon registration	73.76 ± 14.57	0.971 ± 0.008	0.6579 ± 0.129	0.8731 ± 0.0319
VoxelMorph	128.74 ± 32.03	0.9133 ± 0.0266	0.4974 ± 0.1167	0.7856 ± 0.027
RC‐Net	56.42 ± 4.46	0.9814 ± 0.0066	0.6448 ± 0.0717	0.8687 ± 0.0355
RCFRR‐Net	23.44 ± 3.38	0.9972 ± 0.0014	0.8796 ± 0.0406	0.9395 ± 0.0162

Using the quantitative metrics for the registration evaluation, the average RMSE between the moving image and the fixed image was 78.66 ± 13.04 HU while decreasing to 15.54 ± 2.47 HU between the warped image and the fixed image after the proposed RCFRR‐Net registration. The NCC increased from 0.9835 ± 0.0050 to 0.9997 ± 0.0001 and the SSIM increased from 0.7326 ± 0.0430 to 0.8427 ± 0.0460 using the RCFRR‐Net registration.

For the comparison based on ROIs, the RMSE was 336.00 ± 40.13 HU for the ROI‐1, 339.65 ± 37.38 HU for the ROI‐2, 197.99 ± 18.71 HU for the ROI‐3, and 353.17 ± 59.46 HU for the ROI‐4. The RMSE was decreased to 20.12 ± 1.31 HU for the ROI‐1, 21.66 ± 0.69 for the ROI‐2, 16.08 ± 1.34 HU for the ROI‐3, and 23.44 ± 3.38 HU for the ROI‐4 after the proposed RCFRR‐Net registration. The NCC increased from 0.6731 ± 0.0813 to 0.9983 ± 0.0006 for the ROI‐1 and from 0.5964 ± 0.0633 to 0.9975 ± 0.0020 for the ROI‐2. The SSIM increased from 0.2106 ± 0.1121 to 0.8508 ± 0.0613 based on the ROI‐1 and from 0.2319 ± 0.0488 to 0.8978 ± 0.0074 based on the ROI‐2. The Dice score increased from 0.5903 ± 0.0242 to 0.9297 ± 0.038 for the ROI‐1 and from 0.5497 ± 0.086 to 0.9184 ± 0.0177 for the ROI‐2.

### 3.5 Registration evaluation on 4D‐CBCT datasets

**FIGURE 10:**
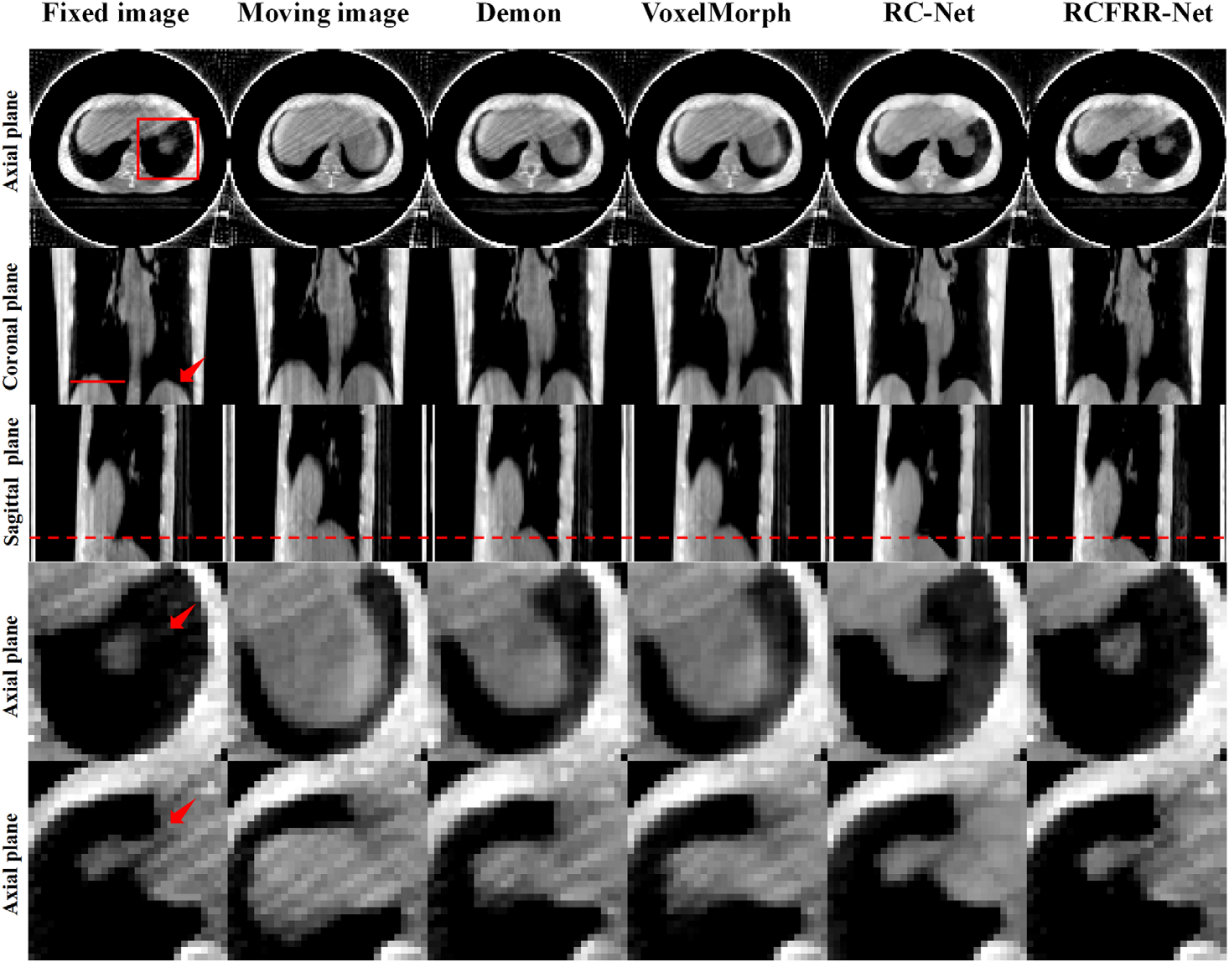
Comparison of the moving images, fixed images, and warped images of different registration methods on 4D‐CBCT datasets. The red dotted lines indicated the organs with large motion. Display window: [−800, 200] HU.

**FIGURE 11:**
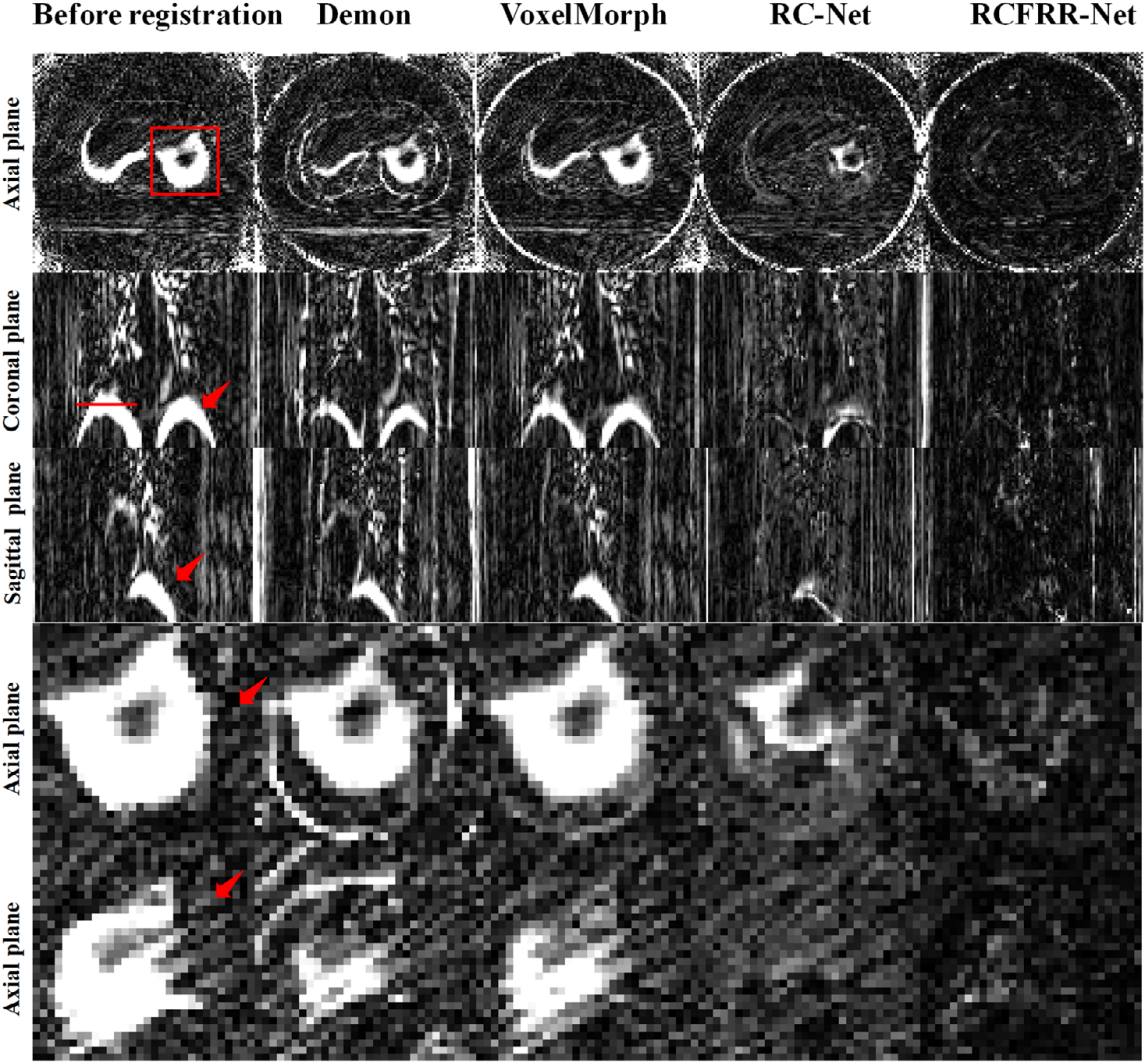
Comparison of the residual images between the fixed images and warped images of different registration methods on 4D‐CBCT datasets. Display window: [0, 400] HU.

Table 6 showed the global image and ROI‐based comparison of the average RMSE, NCC, SSIM, and Dice score using different registration methods for the 4D‐CBCT testing patient. Due to the high level of image noise and artifacts in CBCT images, the registration accuracy was worse than the registration accuracy of CT images. As could be seen from Table 6, the proposed RCFRR‐Net outperformed the other registration methods the lowest RMSE value and the highest NCC/SSIM. Figure 10 presented the axial, coronal, and sagittal planes and the selected ROIs of the moving images, fixed images, and warped images registered by different methods using 4D‐CBCT datasets. The red dotted lines indicated the organs with large motion in the sagittal plane. The absolute difference images between the fixed images and the warped images of different registration methods were shown in Figure 11.

**TABLE 6:** Comparison of RMSE, NCC, SSIM, and Dice score using different registration methods based on 4D‐CBCT datasets.

Comparison content	RMSE (HU)	NCC	SSIM	Dice score
Global image				
Before registration	116.95 ± 34.08	0.9668 ± 0.0164	0.7051 ± 0.1084	–
Demon registration	102.27 ± 28.26	0.9777 ± 0.0109	0.7401 ± 0.0888	–
VoxelMorph	99.73 ± 25.30	0.9769 ± 0.0108	0.7335 ± 0.0983	–
RC‐Net	79.93 ± 14.68	0.9895 ± 0.0032	0.7827 ± 0.0580	–
RCFRR‐Net	56.77 ± 13.22	0.9933 ± 0.0023	0.8607 ± 0.0257	–
ROI‐1				
Before registration	296.25 ± 97.10	0.7850 ± 0.1086	0.4464 ± 0.1596	0.7755 ± 0.0843
Demon registration	183.35 ± 80.21	0.9127 ± 0.0637	0.6041 ± 0.1637	0.8615 ± 0.0441
VoxelMorph	231.18 ± 98.04	0.8701 ± 0.0870	0.5395 ± 0.1745	0.8348 ± 0.0588
RC‐Net	117.04 ± 56.17	0.9711 ± 0.0271	0.7579 ± 0.1052	0.9124 ± 0.0231
RCFRR‐Net	57.80 ± 13.01	0.9919 ± 0.0028	0.8550 ± 0.0427	0.9178 ± 0.0238
ROI‐2				
Before registration	282.10 ± 100.88	0.7767 ± 0.1255	0.4438 ± 0.2053	0.712 ± 0.1007
Demon registration	127.91 ± 51.86	0.9539 ± 0.0328	0.6893 ± 0.1458	0.8361 ± 0.0564
VoxelMorph	182.95 ± 85.87	0.9016 ± 0.0772	0.5860 ± 0.1857	0.7847 ± 0.0781
RC‐Net	85.32 ± 23.99	0.9855 ± 0.0074	0.7734 ± 0.0781	0.8774 ± 0.0385
RCFRR‐Net	55.23 ± 14.42	0.9908 ± 0.0040	0.8797 ± 0.0326	0.8802 ± 0.0368
ROI‐3				
Before registration	282.68 ± 102.69	0.7739 ± 0.1264	0.4845 ± 0.1593	0.7796 ± 0.0825
Demon registration	171.27 ± 74.78	0.9148 ± 0.0635	0.6062 ± 0.1495	0.8434 ± 0.0395
VoxelMorph	218.75 ± 99.59	0.8664 ± 0.0965	0.5606 ± 0.1741	0.8272 ± 0.0545
RC‐Net	112.10 ± 249.22	0.9267 ± 0.0215	0.7403 ± 0.0925	0.904 ± 0.0147
RCFRR‐Net	56.25 ± 12.51	0.9916 ± 0.0028	0.8500 ± 0.0418	0.9124 ± 0.0298
ROI‐4				
Before registration	254.09 ± 81.85	0.8038 ± 0.1055	0.4610 ± 0.1741	0.7692 ± 0.0694
Demon registration	126.38 ± 43.58	0.9494 ± 0.0326	0.6766 ± 0.1285	0.8546 ± 0.0357
VoxelMorph	161.87 ± 69.86	0.9184 ± 0.0612	0.5992 ± 0.1727	0.8189 ± 0.0654
RC‐Net	81.13 ± 18.09	0.9864 ± 0.0048	0.7342 ± 0.0634	0.8906 ± 0.0214
RCFRR‐Net	54.91 ± 13.42	0.9913 ± 0.0038	0.8528 ± 0.0434	0.9024 ± 0.0314

On average, the RMSE between the moving image and the fixed image was 116.95 ± 34.08 HU, while it decreased to 56.77 ± 13.22 HU between the warped image and the fixed image after the proposed RCFRR‐Net registration. The RMSE of the demon registration method, VoxelMorph, and RC‐Net were 102.27 ± 28.26, 99.73 ± 25.30, and 79.93 ± 14.68, respectively. The NCC increased from 0.9668 ± 0.0164 to 0.9933 ± 0.0023, and the SSIM increased from 0.7051 ± 0.1084 to 0.8607 ± 0.0257 based on the RCFRR‐Net. The NCC of the demon registration method, VoxelMorph, and RC‐Net were 0.9777 ± 0.0109, 0.9769 ± 0.0108, and 0.9895 ± 0.0032, respectively. The SSIM of the demon registration method, VoxelMorph, and RC‐Net were 0.7401 ± 0.0888, 0.7335 ± 0.0983, and 0.7827 ± 0.0580, respectively.

The RMSE values for the selected CBCT ROIs were higher than the RMSE values of the selected CT ROIs using the RCFRR‐Net registration. For the selected ROIs of the moving image and the fixed image, the RMSE decreased from 296.25 ± 97.10 HU to 57.80 ± 13.01 HU, from 282.10 ± 100.88 HU to 55.23 ± 14.42 HU, from 282.68 ± 102.69 HU to 56.25 ± 12.51 HU, and from 254.09 ± 81.85 HU to 54.91 ± 13.42 HU, for ROI‐1, ROI‐2, ROI‐3, and ROI‐4, respectively. The NCC, SSIM, and Dice score all had the highest values for the selected ROIs.

## 4 DISCUSSION

In this study, we proposed a novel unsupervised deep learning‐based method for abdominal 4D‐CT image registration. The developed method was tested using multiple datasets (an internal 4D‐CT dataset, a public DIRLAB 4D‐CT dataset, and a 4D‐CBCT dataset). The RCFRR‐Net was trained using the internal 4D‐CT dataset without the DIRLAB 4D‐CT dataset or 4D‐CBCT dataset. The RCFRR‐Net outperformed the comparison methods using all testing datasets. This was a shred of strong evidence that the RCFRR‐Net had a high generalization capability in medical image registration.

The novelty of this study could be summarized in three folds. First, the RCFRR‐Net was trained using the unsupervised approach. The advantage of unsupervised training was that it could alleviate the problem of a lack of ground truth DVFs. Since ground truth DVF was not required, any 4D‐CT dataset could be used for network training. Second, the RCFRR‐Net was constructed using the recursive cascaded method. The similarity was only measured based on the final warped image and the fixed image, allowing all cascades to be trained jointly and perform the progressive registration cooperatively. Third, the cascaded registration subnetwork was developed based on a novel ResNet‐similar architecture, the full‐resolution residual network. The FRRN processed two distinct feature streams: a residual stream and a pooling stream. The residual stream carried feature maps at full resolution with precise boundary information. The pooling stream carried high‐level feature maps with information on image texture. By fusing the two processing streams, both kinds of features carrying image boundary and element information could be obtained simultaneously.

Although the proposed RCFRR‐Net could conduct 4D‐CT image registration with high speed and accuracy, it still needed improvement in future work. The RCFRR‐Net was trained based on the global image, which has a relatively small size. The 3D images were resampled to a size of 96 × 96 × 96. The RCFRR‐Net was not trained using image patches. Because the image patches were usually randomly cropped from the global image, there might be differences in the organizational structure within the paired image patches. This might introduce inevitable errors during the model training process. In the future, we will design an advanced and more appropriate patch extraction method for registration network training. Without increasing the computation resource, the proposed method could achieve image registration at larger sizes.

Since the RCFRR‐Net was trained in a completely unsupervised manner without any prior knowledge about the ground truth, the loss was essential for accurate DVF predictions. In the current study, the loss was composed of similarity in the image domain and smoothness in the DVF domain. The loss was calculated equally for different regions of the global image without considering the physiological motion variance of different regions. In future studies, the physiological motion around the lung could be specifically modeled to accurately capture the complex motion conditions to improve the DIR's performance. For example, the adaptive weights for image similarity and DVF regularization could be incorporated into the loss function.

## 5 CONCLUSIONS

This study presented an unsupervised recursive cascaded architecture for abdominal 4D‐CT DIR. Experiments based on diverse datasets and evaluation metrics demonstrated that the proposed architecture achieved significant gains over state‐of‐the‐art methods. With the superiority of outstanding performance and the general applicability of the unsupervised manner, we expected that the proposed architecture could potentially be extended to more DIR clinical tasks.

## AUTHOR CONTRIBUTIONS

**Lei Xu:** Formal analysis (lead); investigation (lead); methodology (lead); resources (lead); software (lead); validation (lead); visualization (lead); writing – original draft (lead); writing – review and editing (lead). **Ping Jiang:** Writing – review and editing (supporting). **Tiffany Tsui:** Writing – review and editing (equal). **Junyan Liu:** Writing – review and editing (equal). **Xiping Zhang:** Writing – review and editing (equal). **Lequan Yu:** Writing – review and editing (equal). **Tianye Niu:** Data curation (lead); funding acquisition (lead); investigation (lead); methodology (lead); project administration (lead); resources (lead); writing – original draft (lead); writing – review and editing (lead).

## CONFLICT OF INTEREST STATEMENT

The authors declare that they have no competing interests.

### PEER REVIEW

The peer review history for this article is available at https://www.webofscience.com/api/gateway/wos/peer-review/10.1002/btm2.10587.

## Data Availability

The data that support the findings of this study are available from the corresponding author on reasonable request.
